# High post-exposure prophylaxis (PEP) uptake among household contacts of pertussis patients enrolled in a PEP effectiveness evaluation – United States, 2015–2017

**DOI:** 10.1371/journal.pone.0285953

**Published:** 2023-05-18

**Authors:** Lucy A. McNamara, Amy B. Rubis, Lucia Pawloski, Elizabeth Briere, Lara Misegades, Aurora A. Brusseau, Sandra Peña, Karen Edge, Rachel Wester, Kari Burzlaff, Victor Cruz, Lucia Tondella, Tami H. Skoff, for the Pertussis Post-Exposure Prophylaxis Study Team

**Affiliations:** 1 Division of Bacterial Diseases, National Center for Immunization and Respiratory Diseases, US Centers for Disease Control and Prevention, Atlanta, GA, United States of America; 2 New Mexico Department of Health, Santa Fe, NM, United States of America; 3 Colorado Department of Public Health and Environment, Denver, CO, United States of America; 4 New York State Department of Health, Albany, NY, United States of America; 5 Minnesota Department of Public Health, St. Paul, MN, United States of America; LSHTM: London School of Hygiene & Tropical Medicine, UNITED KINGDOM

## Abstract

**Background:**

Post-exposure prophylaxis (PEP) for pertussis is recommended for household contacts of pertussis cases in the United States within 21 days of exposure, but data on PEP effectiveness for prevention of secondary cases in the setting of widespread pertussis vaccination are limited. We implemented a multi-state evaluation of azithromycin PEP use and effectiveness among household contacts.

**Methods:**

Culture- or PCR-confirmed pertussis cases were identified through surveillance. Household contacts were interviewed within 7 days of case report and again 14–21 days later. Interviewers collected information on exposure, demographics, vaccine history, prior pertussis diagnosis, underlying conditions, PEP receipt, pertussis symptoms, and pertussis testing. A subset of household contacts provided nasopharyngeal and blood specimens during interviews.

**Results:**

Of 299 household contacts who completed both interviews, 12 (4%) reported not receiving PEP. There was no evidence of higher prevalence of cough or pertussis symptoms among contacts who did not receive PEP. Of 168 household contacts who provided at least one nasopharyngeal specimen, four (2.4%) were culture or PCR positive for *B*. *pertussis*; three of these received PEP prior to their positive test result. Of 156 contacts with serologic results, 14 (9%) had blood specimens that were positive for IgG anti-pertussis toxin (PT) antibodies; all had received PEP.

**Conclusions:**

Very high PEP uptake was observed among household contacts of pertussis patients. Although the number of contacts who did not receive PEP was small, there was no difference in prevalence of pertussis symptoms or positive laboratory results among these contacts compared with those who did receive PEP.

## Introduction

*Bordetella pertussis* causes pertussis, or whooping cough, a highly contagious respiratory disease with secondary attack rates of up to 80% among susceptible individuals. Introduction of pertussis vaccines during the 1940s significantly decreased the burden of disease in the United States; since the late 1980s, however, the reported number of pertussis cases has gradually increased and epidemic peaks in disease have been reported [1]. Many factors are likely contributing to this reported increase including increased provider recognition, changes in diagnostic testing and reporting, and waning immunity from pertussis vaccines [2].

Periodic evaluation of pertussis prevention and control strategies is critical, especially in the setting of an ongoing pertussis resurgence. Waning immunity from pertussis vaccines is well documented, particularly since the transition to acellular vaccines in the United States during the 1990s [1, 3–8]. Current acellular vaccines have high short-term effectiveness and continue to protect against severe disease; however, many reported pertussis cases in the United States are among fully vaccinated persons [3, 4, 9]. Until improved pertussis vaccines with longer duration of protection are available, alternative pertussis prevention and control strategies such as post-exposure prophylaxis (PEP) can be used alongside vaccination to help control pertussis transmission, especially in the setting of outbreaks among fully vaccinated individuals.

PEP is recommended for many pathogens to prevent secondary cases of disease following exposure to an infected individual [10]. In the United States, pertussis prophylaxis within 21 days of exposure is recommended for household contacts of a confirmed case and for contacts outside the household who are at high risk for severe disease, such as infants <1 year of age [11]; however, implementation of this guidance may vary across U.S. health departments. Macrolide antibiotics, most commonly azithromycin, erythromycin, or clarithromycin, are recommended for pertussis PEP; co-trimoxazole can be used when macrolides are contraindicated [12, 13].

While studies have demonstrated the effectiveness of PEP at preventing secondary transmission of pertussis [14–18], available data have focused primarily on erythromycin which, compared to the newer macrolide azithromycin, is less commonly used because it requires a longer treatment course and has a less favorable side effect profile, resulting in lower compliance [19–22]. It is also unclear whether PEP is needed in the setting of pertussis vaccination, despite waning vaccine-induced immunity [18]. Evaluating the current effectiveness of PEP in preventing secondary pertussis transmission is important to assess whether the benefits of PEP outweigh the potential harms of increased antibiotic use. We therefore implemented a multi-state study with the objective of describing the use of azithromycin PEP and its effectiveness for preventing secondary cases of pertussis among household contacts of U.S. pertussis cases.

## Methods

Culture- or PCR-confirmed pertussis cases with cough of any duration were identified through routine Enhanced Pertussis Surveillance (EPS) as part of the Emerging Infections Program Network between March 1, 2015 and December 31, 2017. The study catchment area included four EPS sites: Colorado (5-county Denver metro area), Minnesota (statewide), New Mexico (statewide), and New York (15 Albany and Rochester area counties). The sites were selected for study participation based on site interest. EPS captures all reported pertussis cases within the catchment area that meet the above case definition; however, only cases reported and interviewed by health department staff within 21 days of cough onset were considered for enrollment. Household contacts were recommended a five-day course of azithromycin PEP based on local and state health department recommendations.

**Fig 1:**
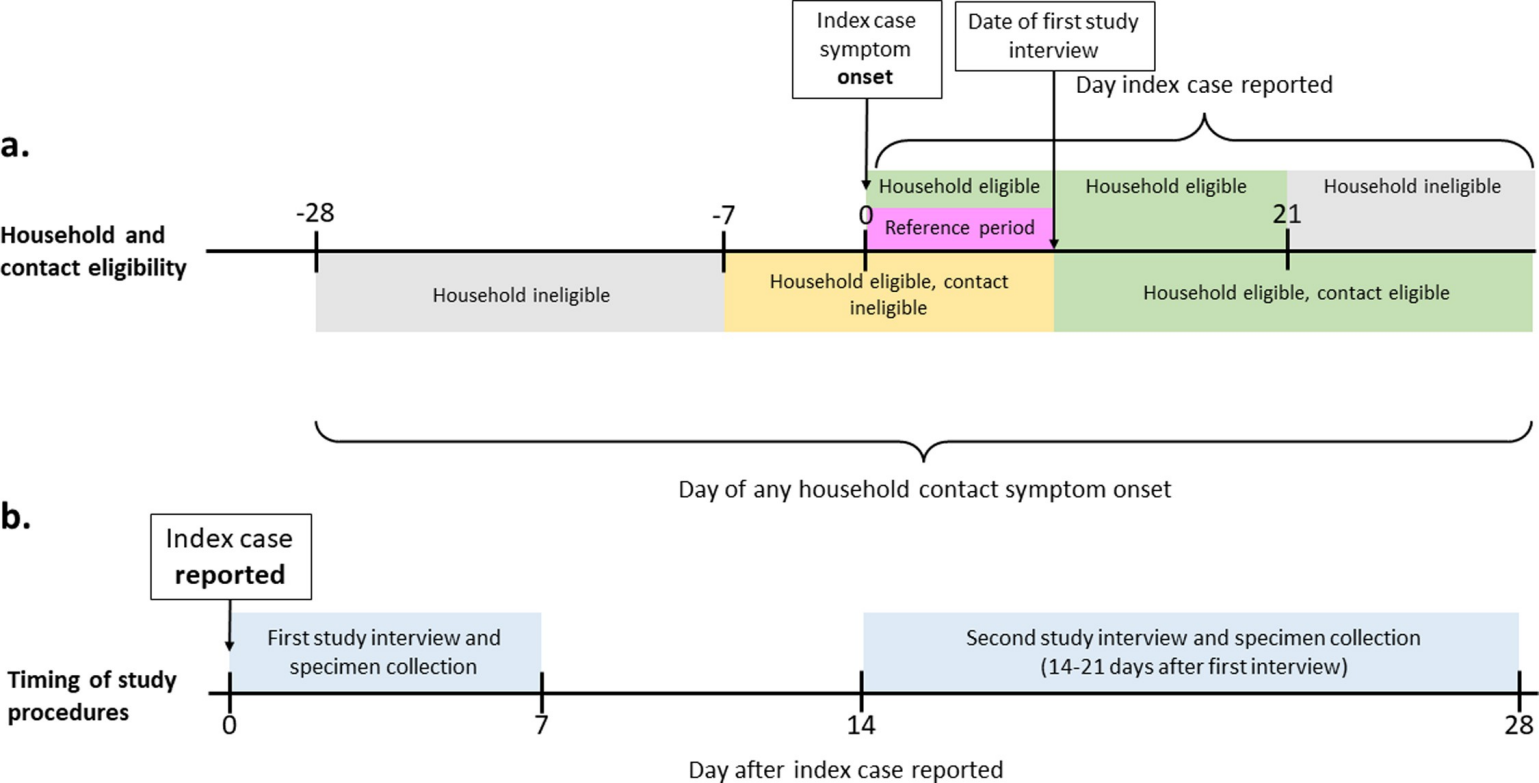
Timeline of household and contact eligibility and implementation of study procedures. **a.** Top, household eligibility based on day that the index case is reported relative to the day of index case cough onset. Bottom, household or individual household contact eligibility based on day of onset of acute cough illness in a household contact relative to day of cough onset in the index case. If any household contact had acute cough illness or pertussis diagnosis with illness onset 7 to 28 days prior to cough onset in the index case, the household is ineligible. If a household contact had acute cough illness with one or more other pertussis symptom and/or a diagnosis of pertussis with onset between 7 days prior to cough onset in the index case and the time of the first study interview, the contact is ineligible but other contacts in the household remain eligible to participate. **b.** Timing of the two study interviews relative to the day the index case was reported to the health department.

Following reporting of a pertussis case, the patient’s household was assessed for study eligibility as part of the public health investigation of the case. Case households were defined as the location where the index case resided at least 50% of the time during the reference period, defined as the time from index case cough onset through the first study interview date (Fig 1A). Households were ineligible if the index case lived alone, the household was previously enrolled for an earlier pertussis case, the health department was unable to contact the case for the initial public health case investigation within 21 days of cough onset, or any household contacts had acute cough illness or a pertussis diagnosis in the 7–28 days prior to index case cough onset (Fig 1A).

Within eligible households, individual contact eligibility was determined by screening household contacts within 7 days of the date the case was reported to the health department. Household contacts were defined as individuals who resided in the same household at least 50% of the time as the index patient during the reference period. Household contacts were ineligible if they could not be screened within 7 days of case report, had an allergy to macrolide antibiotics, had liver disease, or were taking antibiotics other than azithromycin at the time of first study interview. Household contacts were also ineligible if they had acute cough illness with at least one pertussis symptom (paroxysmal cough, whoop, post-tussive vomiting, or apnea) or were diagnosed with pertussis during the reference period (Fig 1A). Of note, if the onset of illness in the household contact was between 7 days prior to cough onset in the index case and the time of the first study interview, the contact was ineligible but other contacts in the household remained eligible to participate (Fig 1A).

Eligible household contacts who were screened and consented to participate were interviewed in person by study staff within 7 days of the date the index case was reported to the health department (Fig 1B). The initial study interview collected relationship and length of exposure to the case in the household with earliest cough onset, age, gender, pertussis vaccine history, history of physician-diagnosed pertussis prior to the reference period, underlying conditions (including immunodeficiencies, chronic respiratory issues, and neuromuscular disorders), and pregnancy status. Participating household contacts could optionally provide a nasopharyngeal (NP) and a blood specimen for laboratory testing for evidence of *B*. *pertussis* infection. NP specimens were collected using NP swabs and placed in Regan-Lowe transport media, then stored in a cooler with ice packs for up to 24 hours before being transferred to the public health laboratory or CDC. Blood was collected through venipuncture or finger stick and left at room temperature to clot, then transferred to the public health laboratory and centrifuged within 24 hours of collection to separate and aliquot the serum. Sera were stored at -40-80°C and batch-shipped frozen to CDC for testing.

At the first study interview, household contacts were given a paper symptom diary (daily symptom checklist) and instructed to record daily information on pertussis symptoms experienced between the first and second study interviews as well as antibiotic adherence for participants who received PEP.

Household contacts were re-interviewed by study staff in person 14–21 days after the initial study interview to capture potential development of pertussis symptoms during most or all of the reported incubation period for pertussis (4–21 days) (Fig 1B). The second study interview collected information on PEP receipt, the number of PEP doses taken, onset of any new pertussis symptoms, and any pertussis testing since the initial study interview. At this time, participants could again optionally provide a NP and blood specimen. However, household contacts were not eligible to have a second set of laboratory specimens collected if they were taking antibiotics other than azithromycin at the time of the second study interview. The symptom diary was returned to study staff at the second visit and was used to validate the symptom questions on the follow-up questionnaire. Participants were classified as having a symptom by the time of the second study interview if the symptom was noted either during the second study interview or on the symptom diary. Household contacts were classified as asymptomatic if they did not report cough or another pertussis symptom at any point during the study period.

NP specimens were tested by culture and PCR. For culture, specimens were plated on Regan-Lowe medium with and without cephalexin and incubated for up to 10 days at 37°C with high humidity; at the New York site, specimens were also plated on Bordet-Gengou media under similar conditions. The PCR assay used by the Colorado, Minnesota, and New Mexico sites was a combination of a multiplex reaction (IS*481*, hIS*1001*, pIS*1001*) and a singleplex reaction (*ptxS1*) that distinguishes among *B*. *pertussis*, *B*. *holmesii* and *B*. *parapertussis* [23]. At the New York site, a multiplex PCR assay targeting IS*481*, BP283, and IS*1001* was used instead. The New York assay also distinguishes *B*. *pertussis* from *B*. *parapertussis* and *B*. *holmesii* [24] and New York State Department of Health unpublished data.

Serum specimens were tested at CDC using an anti-PT IgG ELISA [25]. Samples were tested at a 1:100 dilution and run on two plates with triplicate wells per plate. Final concentration was based on the average of two valid tests. Diagnostic cut-offs were <49 International Units (IU)/mL, 49–93 IU/mL, and ≥94 IU/mL for negative, indeterminate, and positive results, respectively [26]. Specimens collected within 180 days of pertussis vaccination were excluded to ensure that serologic results reflected *B*. *pertussis* infection rather than vaccination [27].

Pertussis vaccine history was verified where possible using participant vaccination cards, immunization registries, or provider records. Age-appropriate pertussis vaccination was defined as described previously [9]. Additional case information, including patient age, sex, pertussis vaccine history, and history of previous pertussis diagnosis, was collected through routine EPS surveillance. Data were analyzed using SAS 9.4. Differences in proportions were tested using Fisher’s exact test; differences in means were assessed using a t-test.

*Patient consent statement*: Written informed consent was obtained from all study participants. The study protocol and informed consent forms were reviewed and approved by the Institutional Review Boards of all participating sites before the start of the study (see 45 C.F.R. part 46; 21 C.F.R. part 56).

## Results

**Fig 2:**
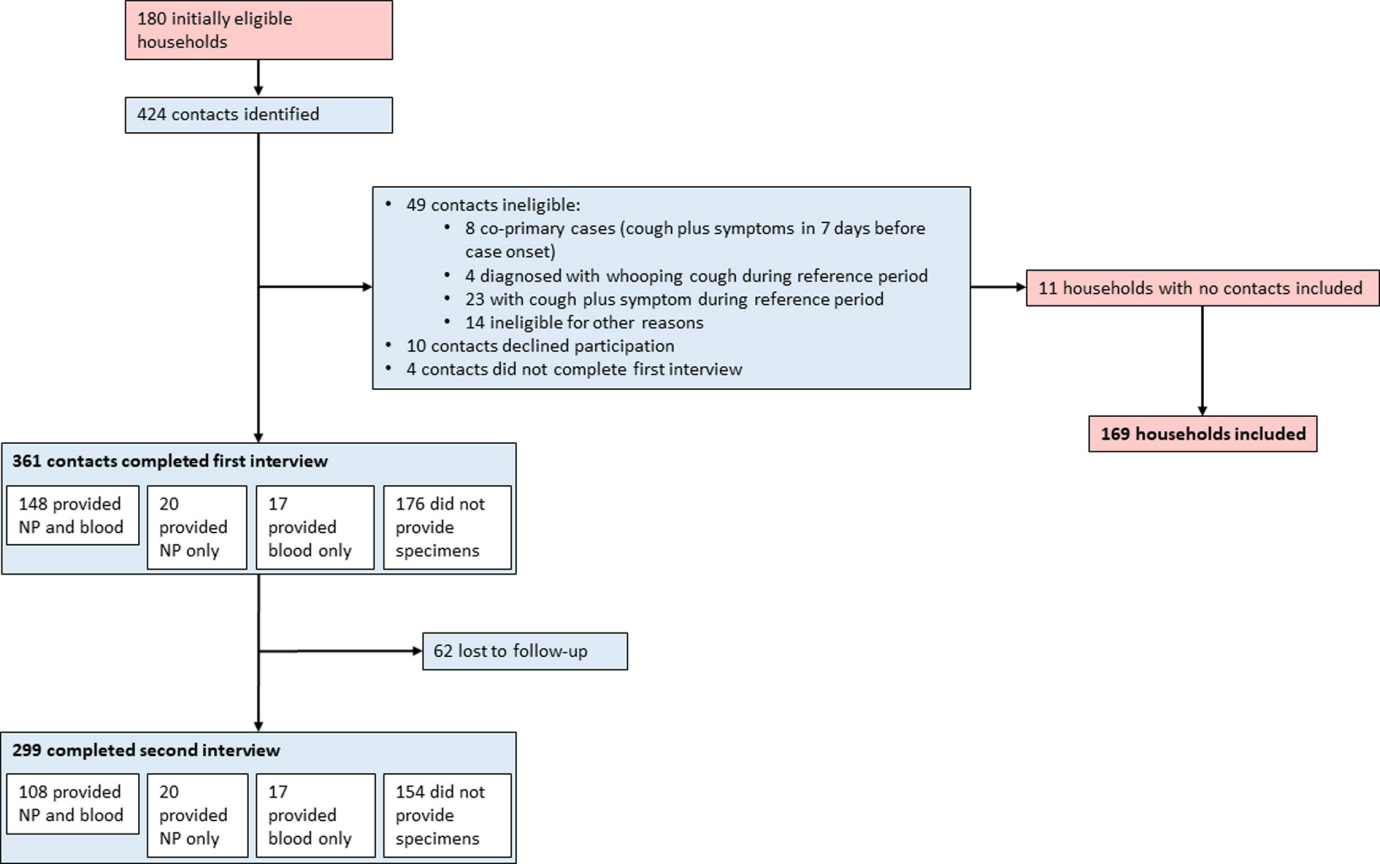
Household contact eligibility, enrollment, and study participation among contacts identified within the 180 initially enrolled households. NP, nasopharyngeal.

Of 2,295 pertussis cases identified in the study catchment area during the study period, 180 pertussis case households (7.8%), which included 424 household contacts, agreed to participate and met household eligibility criteria (Fig 2). Reasons for non-enrollment of identified cases were not systematically captured; however, health departments reported that they were frequently unable to contact index case households within seven days of case notification or and index patients had often been coughing for more than 21 days by the time the household was contacted. In addition, at least 300 eligible households declined to participate.

Among the 424 household contacts in the 180 households that agreed to participate, 49 contacts (12%) were ineligible, most commonly because of pertussis symptoms (31, 7.3%) or diagnosis (4, 0.9%) before the first study interview. Ten additional household contacts (2.4%) declined participation and 4 (0.9%) failed to complete the first study interview, leaving 361 household contacts (85%) from 169 households (94%) who completed the first study interview. Of these 361 household contacts, 168 (47%) provided an NP specimen and 165 (46%) provided a blood specimen at the first study interview. Two hundred ninety-nine (83%) household contacts also completed the second study interview; of these, 128 (43%) provided an NP specimen and 125 (42%) provided a blood specimen at the second study interview.

Most participating households included 1–2 eligible contacts; the majority of participating index cases and household contacts resided in Minnesota and Colorado (Table 1). Over half of index cases were in patients aged 11–18 years, while a majority of household contacts were aged 30–64 years. Approximately one third of household contacts were mothers of the index patients and another third were siblings; fathers and other relatives each accounted for a smaller proportion.

**Table 1:** Index case and household contact characteristics.

	Index cases		First interview contacts		Second interview contacts	
	N	%	N	%	N	%
**Total N**	169	-	361	-	299	82.8
**Eligible contacts per household**			-	-	-	-
1	61	36.1	-	-	-	-
2	58	34.3	-	-	-	-
3	26	15.4	-	-	-	-
4 or more	24	14.2	-	-	-	-
**Age group**						
<1 year	7	4.1	6	1.7	5	1.7
1–6 years	25	14.8	31	8.6	24	8.0
7–10 years	30	17.8	41	11.4	33	11.0
11–18 years	89	52.7	56	15.5	49	16.4
19–29 years	2	1.2	25	6.9	14	4.7
30–64 years	13	7.7	198	54.8	171	57.2
> = 65 years	3	1.8	4	1.1	3	1.0
**Gender**						
Male	76	45.0	147	40.7	120	40.1
Female	75	44.4	212	58.7	178	59.5
Missing	18	10.7	2	0.6	1	0.3
**State**						
CO	47	27.8	119	33.0	105	35.1
MN	103	61.0	203	56.2	158	52.8
NM	7	4.1	16	4.4	13	4.3
NY	12	7.1	23	6.4	23	7.7
**Relationship to case**	-	-				
Mother	-	-	125	34.6	106	35.5
Father	-	-	66	18.3	53	17.7
Sibling	-	-	121	33.5	107	35.8
Other	-	-	48	13.3	33	11.0
Missing			1	0.3	0	0
**Ever received pertussis vaccine**						
Yes	122	72.2	217	83.1	266	89.0
No	9	5.3	15	5.7	12	4.0
Unknown	38	22.5	29	11.1	21	7.0
**Age-appropriate pertussis vaccination**						
Yes	104	61.5	237	65.7	199	66.6
No	20	11.8	36	10.0	30	10.0
Unknown / too young*	45	26.6	88	24.4	70	23.4
**Previous pertussis diagnosis**						
Yes	1	0.59	7	1.9	7	2.3
No	147	87.0	344	95.3	283	94.6
Unknown	21	12.4	10	2.8	9	3.0
Received azithromycin PEP**	-	-				
Yes, <7 days after index case onset	-	-	59	16.3	56	18.7
Yes, 7–13 days after index case onset	-	-	185	51.2	150	50.2
Yes, ≥14 days after index case onset	-	-	84	23.3	75	25.1
Yes, timing unknown	-	-	1	0.3	1	0.3
No	-	-	23	6.4	12	4.0
Unknown	-	-	9	2.5	5	1.7

*Too young <2m old at time of interview (1 case and 1 contact)

**By time of the last study interview the contact participated in

Seventy-two percent of index cases and over 80% of contacts had ever received a pertussis vaccine and 62–67% had received age-appropriate pertussis vaccination. Of note, most remaining participants had unknown vaccination status; only 4–6% reported having never been vaccinated (Table 1). One index case and seven household contacts reported a previous pertussis diagnosis, which occurred 4–45 years prior to the study.

Only 23 (6%) contacts who participated in the first study interview and 12 (4%) who participated in both study interviews reported never receiving PEP (Table 1). The 12 contacts who had not received PEP by the second study interview were aged 1–57 years. Two of these 12 contacts, both adults, had never been vaccinated for pertussis; none of the 12 reported a previous pertussis diagnosis. The 12 contacts were associated with five households. In three of these households (containing one, four, and five eligible contacts), no eligible household contacts received PEP. The remaining two contacts who did not receive PEP were both fathers of the index patient; each belonged to a household where all other contacts (n = 1 or 3) did receive PEP. Only one household contact, the 57-year-old father of an index patient, reported receiving a PEP prescription but not filling it.

Of those who received azithromycin PEP, only three household contacts reported not completing the full five-day course; all three took PEP for four days. The mean time between index case cough onset and contact PEP administration shortened slightly over the study period, with somewhat faster PEP receipt among household contacts aged <6 or ≥65 years compared to household contacts in other age groups (S1 Table). PEP receipt was also faster among household contacts who were pregnant (S1 Table).

By the second study interview, 22% of enrolled household contacts had developed cough, and 6.5% had developed cough with at least one additional pertussis symptom (Table 2). However, among the 12 household contacts who had not received PEP by the second study interview, only one (8.3%) reported cough and none reported cough plus additional pertussis symptoms (Table 2). The prevalence of cough or cough plus additional pertussis symptoms was similar among all PEP recipients regardless of the timing of PEP receipt after cough onset in the index patient.

**Table 2:** Pertussis symptoms and positive or indeterminate nasopharyngeal swab or serologic results among household contacts of pertussis cases, by receipt of post-exposure prophylaxis (PEP).

	Second interview participants					Any nasopharyngeal culture or PCR results available					Any serologic results available				
	Total	Cough		Cough and additional symptom		Total	Positive		Indeterminate		Total	Positive		Indeterminate	
	N	N	%	N	%	N	N	%	N	%	N	N	%	N	%
**Received PEP (days after index case onset)**															
Any	279	63	23	18	6.5*	148	3	2.0	1	0.68	142	14	9.9	14	9.9
<7	54	11	20	3	5.7**	20	0	0	0	0	19	3	16	1	5.3
7-13	150	34	23	10	6.7	81	2	2.5	0	0	76	6	7.9	6	7.9
> = 14	74	18	24	5	6.8***	47	1	2.1	1	2.1	47	5	11	7	15
Unknown	1	0	0	0	0	0	0	--	0	--	0	0	--	0	--
**No PEP**	12	1	8.3	0	0	13	1	7.7	0	0	9	0	0	1	11
**Unknown PEP**	4	1	25	1	25	7	0	0	0	0	5	0	0	1	20
**Total**	295	65	22	19	6.5†	168	4	2.4	1	0.6	156	14	9	16	10

*Of 277 with results available

**Of 53 with results available

***Of 73 with results available

†Of 293 with results available

We next examined vaccination and other factors that were potentially associated with pertussis symptoms among household contacts. While no factors were significantly associated with development of cough alone by the second study interview, household contacts who had never received a pertussis vaccine were significantly more likely to have cough and at least one additional pertussis symptom by the second study interview (S2 Table). There was no significant difference in the mean age of household contacts who did or did not develop cough (27.1 vs. 31.0 years, p = 0.12) or cough and another pertussis symptom (25.8 vs. 30.6 years, p = 0.26) or who were or were not vaccinated (28.4 vs. 32.8 years, p = 0.33).

**Fig 3:**
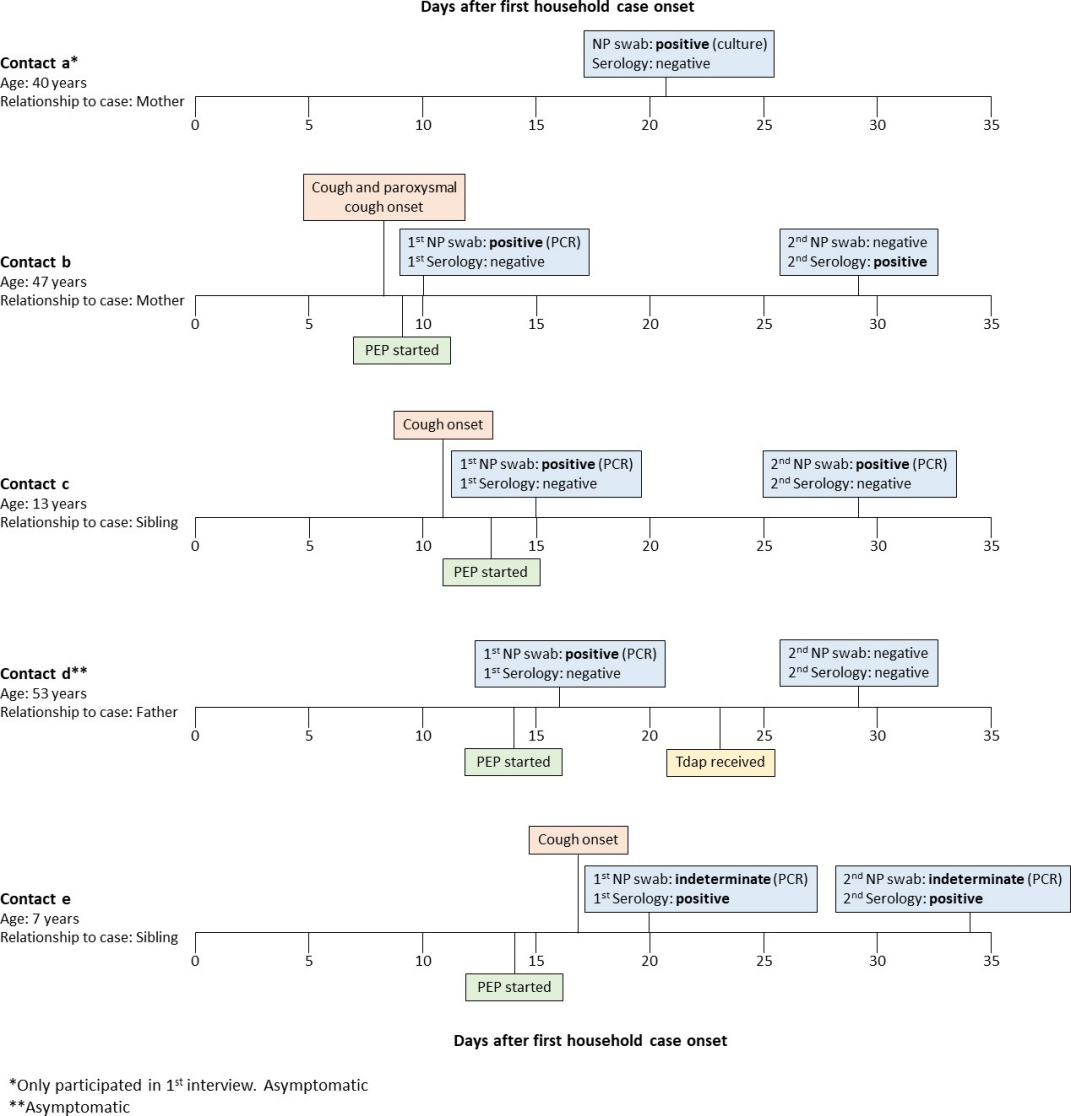
Timing of key events for the five household contacts with positive PCR or culture for *B*. *pertussis* on nasopharyngeal swabs. Contacts **a** and **d** were asymptomatic at all study interviews in which they participated. NP, nasopharyngeal; PCR, polymerase chain reaction; PEP, post-exposure prophylaxis.

Of the 168 household contacts who provided at least one NP specimen, one (0.6%; Fig 3, contact a) was culture-positive for *B*. *pertussis* and three (1.8%; Fig 3, contacts b-d) were PCR-positive at either the first or second study interview. A fifth household contact (Fig 3, contact 3) was indeterminate by PCR at both the first and second study interviews. The culture-positive individual (contact a) had not received PEP at any point; the remaining four received PEP one to six days prior to their positive or indeterminate PCR result. One of the PCR-positive individuals (contact c) had a second positive PCR result 16 days after PEP initiation. None of these five household contacts had any of the underlying conditions (immunodeficiencies, chronic respiratory issues, and neuromuscular disorders) assessed in the study.

Of the four household contacts with positive NP specimens, two (contacts b and c) developed cough, including one with paroxysmal cough. The remaining two household contacts with positive NP specimens (contacts a and d) were asymptomatic at each study interview. One asymptomatic contact (contact a) was culture-positive at the first study interview; however, this individual did not participate in the second study interview and we were therefore unable to assess whether pertussis symptoms developed at a later time point. The second asymptomatic individual (contact d) was PCR-positive at the first study interview but PCR-negative by the second study interview.

Serum specimens from participants were tested for serologic evidence of *B*. *pertussis* infection. Of 156 household contacts who provided blood specimens, 14 (9%) had blood specimens that were positive for IgG anti-PT antibodies at any study interview (Table 2); all had received PEP. Of these, six (43%) had cough and three (21%) had cough with at least one additional pertussis symptom. Among seropositive individuals, there was no significant difference in antibody concentrations between those with or without cough (t-test p = 0.2). Eleven of the 14 individuals with positive serology also provided one or more NP swab specimens; of these, one was positive by PCR at the first study interview but negative at the second, and one was indeterminate by PCR at both study interviews. Interestingly, the individual who was PCR-positive for *B*. *pertussis* at both study interviews was negative for IgG anti-PT antibodies at both time points (Fig 3).

Because very few household contacts had NP swabs that were positive for *B*. *pertussis* by culture or PCR, or serology specimens positive for anti-pertussis toxin antibodies, the relationship between PEP receipt and positive NP or serum specimens could not be assessed.

## Discussion

In concordance with CDC guidance for pertussis prevention and control, we found high and rapid PEP uptake among household contacts of pertussis patients enrolled in our study. While this high PEP uptake was encouraging, especially in an era of pertussis resurgence, it unfortunately limited our ability to address our primary study question, which was to assess the effectiveness of PEP at preventing pertussis among household contacts. However, among the few contacts who did not receive PEP in our study, none had cough with other pertussis symptoms at the time of the second study interview. There was also no difference in prevalence of cough and other pertussis symptoms among those who received PEP earlier vs. later after index case cough onset, suggesting that delays in PEP administration may not increase the secondary transmission of pertussis to household contacts.

Our findings contrast with the results of a recent study conducted in Spain, which demonstrated a significant decrease in secondary attack rate among contacts who received azithromycin PEP within seven days of index case symptom onset [28]. Since the Spanish study included more than five times as many contacts as our study, this difference is likely driven by sample size. Differences between the enrolled study populations may also have contributed; the Spanish study included contacts who resided outside the index patient household and the distributions of contact age, gender, and relationship to the index case also differed. Unfortunately, the potential contribution of differential vaccination coverage to the difference in findings cannot be readily assessed as the Spanish study characterized vaccination status only among contacts ≤18 years of age.

Overall, only 6.5% of enrolled household contacts developed cough with an additional pertussis symptom during this study, and only four (2.4%) of the 168 contacts who provided at least one NP specimen had a positive culture or PCR result for *B*. *pertussis*. This low secondary attack rate is consistent with that observed in the Spanish study [28] and contrasts with previously reported secondary attack rates of 80% or more among unvaccinated household contacts [29, 30], suggesting that pertussis secondary attack rates are reduced in a setting of high PEP and vaccination uptake. While the proportion of household contacts with cough with or without additional symptoms (22%) was higher than the proportion that had cough with an additional symptom, this non-specific presentation likely includes individuals with other respiratory illnesses besides pertussis. Of note, household contacts who had never been vaccinated pertussis had a 25% prevalence of cough and another pertussis symptom at the second study interview, compared with 5.4% among those who had ever received a pertussis vaccine. This finding further underscores the importance of pertussis vaccination despite challenges with waning of vaccine-induced immunity.

The limited evidence for PEP effectiveness in the context of high pertussis vaccination uptake and widespread community transmission supports the U.S. Centers for Disease Control and Prevention stance for limiting pertussis PEP to household and high-risk contacts. Even with targeted PEP administration, there remain concerns that the benefits of PEP may be outweighed by the drawbacks of promoting antibiotic resistance through overuse of azithromycin. Additionally, while infants are at highest risk for severe pertussis morbidity and mortality during the early months of life, before they are old enough to be vaccinated, and therefore arguably might benefit the most from PEP, an association between azithromycin receipt and infantile hypertrophic pyloric stenosis has been reported [31]. Based on the unclear benefits and potential drawbacks of PEP, some countries as well as some U.S. state health departments have implemented more restrictive variations of this pertussis PEP guidance, such as only recommending PEP for high-risk contacts both within and outside the household setting [32].

A major limitation to this study was the extremely low power to detect a difference in pertussis secondary attack rate between household contacts who did or did not receive PEP. Whereas the target sample size to achieve adequate statistical power was 1,424 household contacts, and over 2,000 case households were originally identified during the study time period, only 169 households and 361 contacts were ultimately included in the study. Low enrollment was due in large part to difficulties in identifying and contacting cases within the time limits prescribed by the study; however, the refusal rate was also high, with over 300 eligible households declining to participate. The high refusal rate not only limited the sample size but also the generalizability of our results, as households that refuse to participate may have been less likely to have high uptake of PEP. This concern about generalizability is exacerbated by the fact that a large majority of study participants were recruited from only two states, Colorado and Minnesota. Finally, among households that did participate, PEP uptake was very high and rapid, so the group of individuals who had not received PEP by the first or second study interviews was very small. Considering the high PEP administration rates observed among household contacts in our study sites, further attempts to study PEP effectiveness might be more readily conducted in locations with more restrictive pertussis PEP policies to increase the size of the non-PEP recipient comparison group.

While the very small number of non-PEP recipients precluded our ability to statistically assess the effectiveness of PEP, our study does suggest that the combined strategy of pertussis vaccination and PEP reduces pertussis infection among household contacts to levels far lower than observed historically [29, 30]. The prevalence of pertussis symptoms among household contacts was similar regardless of timing of PEP receipt after index case onset; in contrast, the frequency of cough plus an additional symptom was increased among unvaccinated or inadequately vaccinated household contacts. These findings thus reaffirm that despite the resurgence of pertussis and concerns around waning immunity from vaccines, adhering to pertussis vaccine recommendations remains an effective way to prevent additional cases of pertussis. Meanwhile, further study of PEP effectiveness in a setting of less uniform PEP uptake would help inform the best use of this strategy as the global community continues to wait on the development of next-generation pertussis vaccines.

## Supporting information

S1 Table PEP receipt and timing of PEP administration among household contacts of pertussis cases, by contact characteristics. (DOCX)

S2 Table Development of pertussis symptoms among household contacts of pertussis cases by vaccination status, relationship to case, and timing of case antibiotic treatment. (DOCX)
