# Supplementary material for: High post-exposure prophylaxis (PEP) uptake among household contacts of pertussis patients enrolled in a PEP effectiveness evaluation – United States, 2015–2017
Source: PLoS One. 2023 May 18;18(5):e0285953. doi: 10.1371/journal.pone.0285953 (PMC10194911; doi:10.1371/journal.pone.0285953)
Supplement: S1 Table — (DOCX) [file pone.0285953.s001.docx]

**S1 Table**. **PEP receipt and timing of PEP administration among household contacts of pertussis cases, by contact characteristics**

|  | Received PEP | | No PEP | | Unknown PEP | | Total | Mean days to PEP*, among PEP recipients |
| --- | --- | --- | --- | --- | --- | --- | --- | --- |
|  | **N** | **%** | **N** | **%** | **N** | **%** | **N** |  |
| Year of index case onset |  |  |  |  |  |  |  |  |
| 2015 | 136 | 89 | 16 | 11 | 1 | 0.65 | 153 | 10.8 |
| 2016 | 152 | 92 | 7 | 4.2 | 7 | 4.2 | 166 | 10.7 |
| 2017 | 40 | 98 | 0 | 0 | 1 | 2.4 | 41 | 9.5 |
| Contact age group |  |  |  |  |  |  |  |  |
| <1 year | 6 | 100 | 0 | 0 | 0 | 0 | 6 | 9.5 |
| 1-6 years | 25 | 81 | 6 | 19 | 0 | 0 | 31 | 8.9 |
| 7-10 years | 37 | 90 | 2 | 4.9 | 2 | 4.9 | 41 | 11.0 |
| 11-18 years | 55 | 98 | 0 | 0 | 1 | 1.8 | 56 | 11.3 |
| 19-29 years | 20 | 80 | 2 | 8 | 3 | 12 | 25 | 10.0 |
| 30-64 years | 182 | 92 | 12 | 6.1 | 3 | 1.5 | 197 | 10.7 |
| ≥65 years | 3 | 75 | 1 | 25 | 0 | 0 | 4 | 8.7 |
| Pregnant |  |  |  |  |  |  |  |  |
| Yes | 6 | 86 | 0 | 0 | 1 | 14 | 7 | 8.0 |
| No | 322 | 91 | 23 | 6.5 | 8 | 2.3 | 353 | 10.6 |

*Days from index case onset to PEP administration
