# Supplementary material for: High post-exposure prophylaxis (PEP) uptake among household contacts of pertussis patients enrolled in a PEP effectiveness evaluation – United States, 2015–2017
Source: PLoS One. 2023 May 18;18(5):e0285953. doi: 10.1371/journal.pone.0285953 (PMC10194911; doi:10.1371/journal.pone.0285953)
Supplement: S2 Table — (DOCX) [file pone.0285953.s002.docx]

**S2 Table. Development of pertussis symptoms among household contacts of pertussis cases by vaccination status, relationship to case, and timing of case antibiotic treatment**

|  | Total | Cough at second interview | | | Cough and additional symptom at second interview | | |
| --- | --- | --- | --- | --- | --- | --- | --- |
|  |  | **N** | **%** | **p*** | **N** | **%** | **p*** |
| Ever received pertussis vaccine |  |  |  |  |  |  |  |
| Yes | 263 | 55 | 21 | 0.29 | 14 | 5.4† | 0.03 |
| No | 12 | 4 | 33 |  | 3 | 25 |  |
| Age-appropriate vaccination with pertussis vaccine** |  |  |  |  |  |  |  |
| Yes | 196 | 39 | 20 | 0.23 | 10 | 5.2†† | 0.10 |
| No | 30 | 9 | 30 |  | 4 | 13 |  |
| Relationship to case |  |  |  |  |  |  |  |
| Mother | 103 | 24 | 23 | 0.31 | 5 | 4.9 | 0.77 |
| Father | 52 | 9 | 17 |  | 3 | 5.8 |  |
| Sibling or step-sibling | 106 | 28 | 26 |  | 9 | 8.6¥ |  |
| Other | 33 | 4 | 12 |  | 2 | 6.1 |  |
| Days to antibiotic treatment of case |  |  |  |  |  |  |  |
| <7 days | 72 | 19 | 26 | 0.86 | 4 | 5.6¥¥ | 0.91 |
| 7 - 13 days | 104 | 23 | 22 |  | 9 | 8.74£ |  |
| 14 - 20 days | 39 | 9 | 23 |  | 3 | 7.7 |  |
| Never treated | 43 | 12 | 28 |  | 3 | 7.0 |  |

* p-values from Fisher’s exact test

† Of 261 with results available

** Excludes one individual aged <2 months (0 doses required for age-appropriate vaccination)

†† Of 194 with results available

¥ Of 105 with results available

¥¥ Of 71 with results available

£ Of 103 with results available
